# Evidence-Based Chinese Medicine for Hypertension

**DOI:** 10.1155/2013/978398

**Published:** 2013-06-03

**Authors:** Jie Wang, Xingjiang Xiong

**Affiliations:** Department of Cardiology, Guang'anmen Hospital, China Academy of Chinese Medical Sciences, Beijing 100053, China

## Abstract

Hypertension is an important worldwide public -health challenge with high mortality and disability. Due to the limitations and concerns with current available hypertension treatments, many hypertensive patients, especially in Asia, have turned to Chinese medicine (CM). Although hypertension is not a CM term, physicians who practice CM in China attempt to treat the disease using CM principles. A variety of approaches for treating hypertension have been taken in CM. For seeking the best evidence of CM in making decisions for hypertensive patients, a number of clinical studies have been conducted in China, which has paved the evidence-based way. After literature searching and analyzing, it appeared that CM was effective for hypertension in clinical use, such as Chinese herbal medicine, acupuncture, moxibustion, cupping, qigong, and Tai Chi. However, due to the poor quality of primary studies, clinical evidence is still weak. The potential benefits and safety of CM for hypertension still need to be confirmed in the future with well-designed RCTs of more persuasive primary endpoints and high-quality SRs. Evidence-based Chinese medicine for hypertension still has a long way to go.

## 1. Introduction

In global health politics, cardiovascular disease is the elephant in the room; it is a massive problem that few want to acknowledge and even fewer want to tackle [1]. Cardiovascular disease (CVD) is as the leading cause of death worldwide, accounting for an estimated 30% and 10% of all deaths and disability, respectively [2, 3]. It is reported that, approximately 62% of strokes and 49% of myocardial infarctions are caused by high blood pressure (BP) [4]. Hypertension is an important worldwide public-health challenge because of its high frequency and concomitant risks of cardiovascular and kidney disease [5]. It affects about 972 million adults worldwide [5] and is attributable each year for 7.6 million excess deaths and loss of 92 million disability-adjusted life years (DALYs) [2]. The purpose of antihypertensive treatment is to prevent the occurrence of CVD, by means of strict control of BP [6]. However, hypertension in most adults remains untreated or uncontrolled. BP control in the population is far from optimal, and SBP/DBP values <140/90 mmHg are achieved in no more than 25% of patients with treated hypertension worldwide [1]. Effective treatment of hypertension is limited by availability, cost, and adverse effects of antihypertensive medications [6]. Thus, due to the limitations and concerns with current available hypertension treatments, a certain proportion of the population, especially in Asia, has turned to complementary and alternative medicine (CAM) [7–11], including Chinese medicine (CM) [12–15], in searching for a treatment modality with potential efficacy and few advice effects. CAM is becoming increasingly popular and frequently used among patients with CVD, but these therapies lack demonstrated efficacy and safety for treating cardiovascular disease including hypertension [16]. Further research is essential in all areas of CAM to confirm its usefulness as an adjunct therapy [17, 18].

Chinese medicine, a system of ancient medical practice that differs in substance, methodology, and philosophy to modern medicine, plays an important role in health maintenance for the peoples of Asia and is becoming more frequently used in countries in the West [19]. It has been used to treat symptoms related to hypertension for more than 2500 years [20, 21]. Today, CM is commonly used to treat hypertension in China and the West [22–25]. And until now, the efficacy of CM for treating hypertension is suggested by a large number of published case series and uncontrolled trials [26–30]. Six randomized controlled trials [31–36] reported significant reductions in BP relative to randomly assigned control groups treated for 4 to 12 weeks, whereas the other six trials [37–42] reported negative results of CM relative to control subjects. For seeking the best evidence of CM in making decisions for hypertensive patients, a number of clinical studies have been conducted in China to gain credibility with the researchers' unremitting efforts. Thus, it is helpful to review the current research status of clinical study of evidence-based Chinese medicine for hypertension. 

The purpose of the paper is to review multiple approaches of Chinese medicine therapies for the treatment of hypertension. The literature available through both English and Chinese search engines that discusses the potential uses of Chinese medicine therapies to treat hypertension is reviewed. The English language literature is searched through the Cochrane Central Register of Controlled Trials (CENTRAL) in the Cochrane Library (September, 2012), MEDLINE (1959–2012), PUBMED (1959–2012), and EMBASE (1980–2012) databases. The Chinese language literature was searched through Chinese National Knowledge Infrastructure (CNKI) (1980–2012), Chinese Scientific Journal Database (VIP) (1989–2012), Chinese Biomedical Literature Database (CBM) (1978–2012), and WANFANG (1998–2012) databases. The following search terms were used individually or combined: “traditional Chinese medicine,” “Chinese medicine,” “Chinese herbal medicine,” “herb,” “blood pressure,” “hypertension,” “essential hypertension.” Chinese terms that were used in search were equivalent to those used to search from English language databases. Finally, 15 systematic reviews (SRs) and meta-analysis [43–57] were collected and reviewed for this study, with 5 articles in English [47, 53–56] and 10 articles in Chinese [43–46, 48–52, 57]. To our knowledge, this is the first systematic English review of the evidence-based Chinese herbs for the treatment of hypertension.

## 2. The Understanding of Hypertension from the Perspective of Chinese Medicine

Different from Western medicine (WM), Chinese medicine (CM) has formed a unique way to diagnose and treat diseases [58]. Great efforts have been made by China's ancient ancestors through meticulous observation of nature, the cosmos, and the human body. And a series of traditional medical practices were originated in China including Chinese herbal medicine (CHM), acupuncture, moxibustion, cupping, qigong, Tai Chi (shadow boxing exercise), diet, and exercise therapy. 

As we know, blood pressure is the diagnostic gold standard in conventional medicine. Thus, there is no concept and diagnosis of hypertension in ancient China. Although hypertension is not a CM term, physicians who practice CM in China attempt to treat the disease using CM principles. According to the typical signs and symptoms of the disease, it falls into the category of “vertigo or headache” in CM [21]. CM has long been used to treat hypertension-related symptoms in clinical practice for centuries. CM approaches hypertension as it does for other diseases under the guidance of holistic concept and treatment based on syndrome differentiation and formula syndrome differentiation [59, 60]. CM has been widely used to certain syndromes and formula syndromes in hypertension, such as fire syndrome, *Banxia Baizhu Tianma Tang* (decoction of *Pinellia ternata*, *Atractylodes*, and *Gastrodia elata*) syndrome [61]. Physicians who prescribe Chinese herbs and formulas recently realized that patient with hypertension exhibit the same pathological changes as those that are characteristic of fire syndrome and *Banxia Baizhu Tianma Tang* (Decoction of *Pinellia ternata*, *Atractylodes *and *Gastrodia elata*) syndrome. Moreover, increasing evidence indicates that, Chinese herbs and formulas that improve fire syndrome and *Banxia Baizhu Tianma Tang* (Decoction of *Pinellia ternata*, *Atractylodes*, and *Gastrodia elata*) syndrome are useful in treating hypertensive patients in China [21].

In our previous studies, hypertension could be divided into the following three major types on the basis of the stage and symptoms of the disease in CM. The first one is fire syndrome which could be found in various stages of hypertension. It can also be further divided into four types such as liver fire, heart fire, stomach fire, and intestinal fire. The second one is phlegm-fluid retention syndrome which often appears in the later stage of the disease. In light of the disease location, it could be divided into three types such as fluid retention in up *jiao* syndrome, fluid retention in middle *jiao* syndrome, and fluid retention in down *jiao* syndrome. The last one is deficiency syndrome. The most common deficiency syndromes are spleen deficiency syndrome and kidney deficiency syndrome. The recommended treatment program of hypertension by Chinese herbal formulas is shown in Table 1.

A variety of approaches for treating hypertension have been taken in CM. Among them, Chinese herbal therapy is the most commonly used. Furthermore, acupuncture, moxibustion, cupping, qigong, Tai Chi (Shadow boxing exercise), and CM external therapy (including bath foot, acupoint application, and thorn collaterals bloodletting) could also be used in the treatment of the disease. 

## 3. Paving the Way for Evidence-Based Chinese Medicine for Hypertension

Evidence-based medicine (EBM), a new paradigm for medical practice, quickly developed in the 1990s. According to the book of “Evidence-based Medicine: How to Practice and Teach EBM” written by Dr. Sackett, one of the pioneers in EBM, EBM makes the explicit, judicious, and conscientious use of the best evidence in making decisions for preventing diseases, promoting the recovery and improving life quality [62]. It has brought great impacts on the efficacy and safety of previous widely accepted strategies of therapeutic, rehabilitative, and preventive regimens by the evidences from a series of systematic reviews and meta-analysis. That is to say clinical experience is unreliable and all medical interventions should be based on rigorous research evidences [63]. Eugene Braunwald, a famous cardiologist, also advocated that current cardiology practice should be evidence based and global in scope [64].

There is close relationship between CM and EBM [65]. Due to the shortage of objective and quantitative criteria in evaluating therapeutic effect and safety in CM, it is urgent to formulate a scientific way. The emergence of EBM had just provided an appropriate method to solve this critical issue. As the applications of CM in the treatment of hypertension are increasing, more and more concern on the efficacy and safety are aroused [22]. Whether CM is equal or superior to WM, how CM plays the role in enhancing efficacy and reducing toxicity, and how to optimize the therapeutic regimen by combination of CM and WM, all these problems are not clear currently. All of these issues warrant further investigation and need more evidences. Here, the paper reviews the background of CM for the treatment of hypertension. 

According to historical records in CM classics, the earliest evidence of Chinese herbal medicine used in China is of two graves from the Han Era (206 B.C. to 220 A.D.) [66]. There are a large number of clinical trials about classical famous prescriptions for the treatment of “vertigo or headache” since ancient time. Although patients with “vertigo or headache” may not necessarily be fully consistent with the diagnosis of hypertension, previous widely used formulae still have a good clinical effect in the treatment of hypertension today. Physicians in ancient China realized that there is a certain connection between a special pattern and a corresponding herb or formula in the clinical practice. And they recorded the treatment process. It is considered as the “clinical trial” in ancient time. After then, the “clinical trial” was tested and repeated by the successors for hundreds or even thousands of years. Thus, it is a unique clinical trial. In the trial, the special pattern is also known as “formula syndrome” or “herb syndrome” [60], which is the indication of Chinese herbs and formulas [59]. The corresponding formula is called classical formulae. These classical formulae in the treatment of hypertension included *Tianma Gouteng Yin *(decoction of *Gastrodia *and *Uncaria*), *Banxia Baizhu Tianma Tang *(decoction of *Pinellia ternata*, *Atractylodes *and *Gastrodia elata*), *Longdan Xiegan Tang *(decoction of radix gentianae for purging liver fire),* Da Chai Hu Tang *(Major *Bupleurum *Decoction), *Zexie Tang *(Decoction of American water Plantain), and *Liu Wei Dihuang Wan *(Pill of *Rehmannia*) [21].

**Figure 1:**
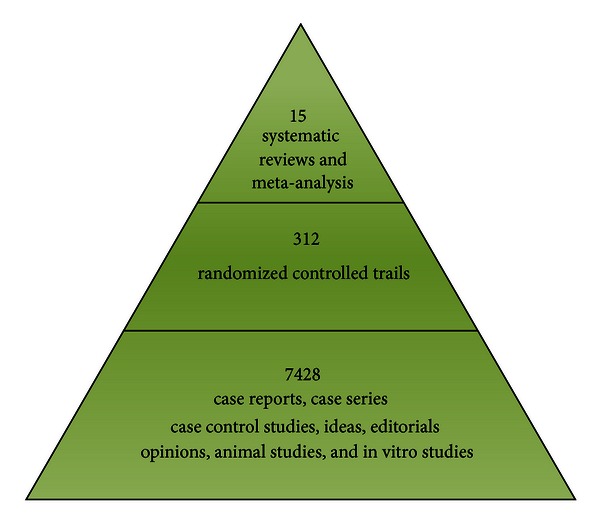
Number of studies on CM for hypertension published in Cochrane Library, PubMed, EMBASE, CNKI, VIP, CBM, and WANFANG.

In order to obtain supportive evidence of CM herbs and approaches that are thought to exert hypotensive effect, we retrieved the data primarily via the Internet (Cochrane Library, PubMed, EMBASE, CNKI, VIP, CBM, and WANFANG) up to September 30, 2012. All the case reports, case series, case control studies, ideas, editorials opinions, animal studies, in vitro studies, randomized controlled trials, systematic reviews, and meta-analysis based on CM for essential hypertension were included. There were no restrictions on population characteristics, language, and publication type. Duplicated publications reporting that the same groups of participants were excluded. We could see from Figure 1 that there are a large number of researches in the field of CM for hypertension in the past 50 years, especially in the past 30 years. 

As shown in the first stage, 7428 studies reported the effectiveness of CM for hypertension ranging from case control studies, case reports, case series, ideas, editorials, opinions, animal studies, and in vitro studies to controlled observational studies. Among the herbal therapies, it includes three categories of formulae. The first one is individually prescribed decoctions, which have been used in most outpatients and inpatients. The second one is currently effective practice formula or experienced prescriptions from famous CM doctors. The third one is the modified classical formulae. The last one is the classical formulae or well-known Chinese medicine formula. In recent decades, the proprietary Chinese medicines (PCMs) for treatment of hypertension are mainly originated from the last three categories. The PCMs have been tested in a large number of clinical trials. 

With increasing awareness and practice of EBM, 312 randomized controlled trials (RCTs) have been conducted to evaluate the effectiveness of CM for hypertension as shown in the second stage.

Regarding clinical effect and its evaluation in clinical researches on CM for hypertension, systematic reviews (SRs) and meta-analysis are important approaches to get the best available evidence. In the third stage of Figure 1, there are 15 SRs and meta-analysis [43–57] collected after literature searches. 

## 4. Exploring the Differences in Response to Treatment from SR

As shown in Table 2, there are 15 SRs and meta-analysis of CM for hypertension published in Chinese or English. Here, these published findings are analyzed to explore the range and role of CM for the treatment of hypertension.

### 4.1. Chinese Herbal Medicine

There are 9 SR of Chinese herbal medicines published whether on individually prescribed decoctions or classical formulae for hypertension. The results are shown in Table 1 [43–51]. Comprehensive evaluations of the clinical efficacy of the Chinese herbal medicine were conducted in 3 SRs [43–45]. Chinese herbal medicine, which could clear fire, suppress liver yang hyperactivity, remove blood stasis, fluid and phlegm, nourish kidney, and reinforce spleen *qi*, were all included in the analysis. The result showed that Chinese herbal medicine the use of alone may be beneficial to reduce BP in patients with hypertension, and no significant difference was found between CM and WM [44, 45]. Combination therapies, just CM combined with WM, showed better results than those of WM for treating hypertension [45]. However, another SR reported negative results that total effective rate and efficiency of CM are lower than that of single WM as for BP controlling [43]. Owing to the lack of data from high-quality RCT, the efficacy needs to be further studied [43–45].

Tianma Gouteng Yin Formula (TGYF), a famous prescription noted in *Za Bing Zheng Zhi Xin Yi* (New Meanings in Syndrome and Therapy of Miscellaneous Diseases), contains eleven commonly used herbs (*Gastrodia Elata, Uncaria, Abalone Shell, Eucommia Ulmoides Oliv, Achyranthes Root, Loranthus Parasiticus, Gardenia, Scutellaria Baicalensis Georgi, Leonurus Japonicus, Poria Cocos*, and caulis polygoni multiflori). It could suppress liver yang hyperactivity, clear heat, activate blood, and nourish the kidney; it has been widely used to treat hypertension-related signs and symptoms in clinical practice for centuries in China. 2 SRs [46, 47] assess the efficacy and safety of TGYF for treating primary hypertension. One SR [46] showed that TGYF combined with enalapril showed additional better effects than enalapril for hypertension. No serious adverse event is reported. However, the other SR could not find any randomized controlled clinical trials that compared TGYF to placebo or no treatment [47]. Authors advised that well-designed randomized controlled studies need to be conducted and published.

Liver yang hyperactivity syndrome, kidney deficiency syndrome, and spleen deficiency syndrome are very common in CM. Aiming to improve these different syndromes, treatment principles of calming the liver and replenishing kidney and spleen were used, respectively. 3 SRs [48–50] assess the efficacy and safety of treatment based on Chinese medicine principles for hypertension. All SR showed curative effect and high safety of CM. 

When referring to elderly isolated systolic hypertension, it indicated that CM is effective on treating isolated systolic hypertension of the old, as well as reducing symptoms and pulse pressure [51]. Authors also gave conclusions that the evidence for the favorable results in the trials is limited, and these findings should be carefully interpreted due to the low methodological quality. 

### 4.2. Acupuncture

Acupuncture is considered an ancient practice of TCM that began thousands of years ago. It has been reported to have potential effectiveness for treating cardiovascular diseases including hypertension, with few reported adverse effects [67, 68]. Several features of acupuncture make it an attractive therapeutic alternative with increasing popularity [36]. Although the results of the trials showed a tendency that acupuncture can improve the conditions of essential hypertension, a reliable conclusion cannot be drawn from the present data because of the defects in methodological quality and insufficient numbers of trials [52]. Thus, evidence of efficacy in lowering blood pressure from controlled trials has been scant. It is necessary to perform more multicentral RCTs of high quality in the future.

### 4.3. Moxibustion

Moxibustion, a traditional medical intervention of CM, involves the application of ignited mugwort (*Artemisia vulgaris*) directly or indirectly at acupuncture points or other specific parts of the body to treat or prevent diseases [69]. The mechanism of moxibustion maybe related to the combination of heat (burning pain and heat stress), tar (extract), aroma (fume), and psychological stress [70]. According to the theory of CM, a possible explanation for how moxibustion works is that heat could increases *qi *circulation and relieves *qi *stagnation by stimulating the acupuncture points to regulate the function of meridians and visceral organs [71]. A SR on the effects of moxibustion on hypertension revealed no evidence that moxibustion is beneficial to people with hypertension [53]. Differences between specific and nonspecific effects should be examined in a future study, and rigorously designed trials are warranted to answer the many remaining questions. 

### 4.4. Cupping

Cupping therapy, as a part of CM, is widely used in treating pain and many other complaints for millennia [72]. A glass cup is utilized to create suction over a painful area or an acupuncture point after incisions are made to the skin. By doing so, the skin is pulled into the cup without drawing blood. Therefore, negative pressure acts on the skin and irritates subcutaneous muscles. It is often used to lower BP and relieve hypertension-related symptoms such as headaches and anxiety [73]. A SR on the effect of cupping on hypertension revealed no significantly convincing evidence to suggest that cupping is effective for treating hypertension. Further research is required to investigate whether it generates any specific effects for that condition [54]. 

### 4.5. Qigong

Qigong, as an ancient Chinese healing art, is widely used in Asia and has been officially recognized as a standard medical technique in Chinese hospitals. It involves exercises for posture, coordination of different breathing patterns, movement, and meditation [74]. According to the theory of CM, it could increase the healthy flow of *qi* throughout the body to heal itself. It is claimed that qigong has potential beneficial effects on various disorders, including cardiovascular disease [75]. Several RCTs have claimed that qigong has therapeutic effects on blood pressure in patients with hypertension [76–78]. 2 SRs on the effects of qigong on hypertension revealed some encouraging evidence of qigong for lowering BP. However, the conclusiveness of these findings is limited. Rigorously designed trials are warranted to confirm these results [55, 56].

### 4.6. Tai Chi

Tai Chi (also known as Tai Chi Quan or Shadow Boxing), originated in ancient China, is a Chinese conditioning exercise well known for its graceful movement. It has been practiced for centuries in the East for health promotion and longevity. In recent years, there has been a growing interest and prevalence in Tai Chi exercise in western societies. During the practice, it combines deep diaphragmatic breathing with continuous body motions to achieve a harmonious balance between body and mind. Previous researches have indicated that Tai Chi exercise may improve health-related fitness (including cardiorespiratory function, muscular strength, balance, and flexibility), quality of life, and psychological well-being. Recent studies also suggest that it may have beneficial effects for patients with cardiovascular conditions and some cardiovascular risk factors [79, 80], including hypertension [81]. There are few trials on the effectiveness of Tai Chi in the management of hypertension. A SR [57] including 5 randomized clinical trials with 318 hypertensive patients reported some positive findings for Tai Chi on treating essential hypertension. It is also pointed out that different exercise time of Tai Chi has an impact on hypertension. However, more RCTs of high quality are warranted to prove benefits of Tai Chi on hypertensive patients with different stages.

## 5. Providing Evidence of Safety

It is widely accepted that herbal medicine is undoubtedly safe for various diseases in China. However, with the increasing reports of liver toxicity and other adverse events associated with Chinese herbal medicines [82–84], the safety of CM needs to be monitored rigorously and reported appropriately in the future clinical trials [85–87]. According to our review, safety evaluation of CM is not the highlight. Inadequate reporting on adverse events is found either in the included SRs or in the original RCTs. Most of the adverse effects of CM were mentioned as “low adverse effect” or “none obvious.” Only six of the fifteen SRs reported the adverse effect of CM briefly, providing limited information [43, 46, 48–50, 56]. Among them, four SRs about Chinese herbal medicine [43, 46, 48, 50] reported nine specific symptoms in treatment group including headache, dizziness, dry mouth, dry cough, abdominal distension, and constipation. However, all these adverse events could be tolerated by participants in the trials. The other two SRs reported no serious adverse event in replenishing kidney *qi* group [49] and qigong group [56]. The rest nine SRs did not mention whether they had monitored adverse effects at all [44, 45, 47, 51–55, 57]. It is generally believed that acupuncture, moxibustion, cupping, qigong, and Tai Chi have reliable safety. Unfortunately, included SRs about these approaches provided insufficient evidence. Therefore, conclusions about the safety of CM cannot be made from this paper and needs to be further proven, due to the limited, inadequate recording, and reporting of adverse events. 

## 6. Discussion

Considerable progress has been made by domestic and foreign clinical experts and researchers for the treatment of hypertension [88–90]. Oral antihypertensive drugs are a milestone in the therapy of essential hypertension. However, the current awareness, control, and mortality rates of hypertension are still far from optimal [91, 92]. Only 25% of patients could achieve the goal, and recurrent cardiovascular events still occur in those who take antihypertensive drugs. What's more, numerous adverse reactions, including headache, dizziness, orthostatic hypotension, and decreased sexual function, limit the clinical practice of antihypertensive drugs [93]. Thus, traditional medicine (TM) has got increasing popularity with people all over the world [94–97]. CM has made great contributions to the health and well-being of the people for its unique advantages in preventing and curing diseases, rehabilitation, and health care [98–103]. Over the past 30 years, the study of CM for treating hypertension is the most active area of researches worldwide [104–108]. Significant progress has been made from theory and experiments to clinic fields based on the inheritance and innovation of thoughts of CM for hypertension [109–113]. A recent study investigated the multiprotective mechanisms of Chinese herbal formulas for treating hypertension from the perspective of modern science, including smoothly controlling BP, reducing blood pressure variability (BPV), protecting target organs, regulating renin-angiotensin-aldosterone system (RAAS), reversing risk factors, improving endothelial function, blocking calcium channels, improving life quality and clinical symptoms, and reversing uncontrollable factors of BP [114]. Therefore, much attention has been paid to the holistic, multitarget, and multidimensional pharmacological studies of CM currently.

The systematic reviews indicated the potential benefit of CM for hypertension in terms of some outcome measures, but none of them drew a definite conclusion due to the poor quality of primary studies. Poor methodology and reporting quality of SRs about CM have caused widespread concern [115, 116]. According to preferred reporting items for systematic reviews and meta-analyses (PRISMA) statement, it was found that most of the included reviews have poor quality [117]. If the reviews are poorly designed and reported, misleading conclusions about current clinical practice would be given. So, the reporting of SR should be in accordance with PRISMA in future researches. The included literature must be scrutinized and selected strictly in order to avoid the potential bias. Process of extracting data such as study design, allocation sequence, allocation concealment, blinding, intention to treat analysis, and drop outs should be conducted rigorously. Also, how to evaluate the validity of the primary studies is an important aspect. It is well known that the primary goal of the treatment for essential hypertension is to reduce mortality and prevent progression to heart disease and other complications of hypertension. The secondary endpoints are mainly blood pressure, blood liquid, and traditional Chinese medicine syndromes [118]. Our overview showed that there is a lack of definite data on the primary endpoints, whereas the secondary endpoints were most commonly adopted in clinical trials. Therefore, the persuasion of conclusions about CM for hypertension would be reduced greatly. Although it appeared that CM was effective for hypertension in clinical use, such as Chinese herbal medicine, acupuncture, moxibustion, cupping, qigong, and Tai Chi, most SRs were inconclusive that CM had a definite effect owing to the poor evidence. As weak recommendations result from low quality evidence, high quality evidence of CM for hypertension is warranted in further RCTs to guide clinical practice either for hypertensive patients or physicians. 

In conclusion, evidence-based Chinese medicine for hypertension still has a long way to go [119, 120]. The potential benefits and safety of CM for hypertension still need to be confirmed in the future with well-designed RCTs of more persuasive primary endpoints and high-quality SRs. Although the development of evidence-based CM for hypertension will be full of challenge, we have full confidence. 

## Figures and Tables

**Table 1:** Recommended treatment program of hypertension by Chinese herbal formulas.

Syndrome	Clinical signs	Treatment principles	Classical formula
Fire syndrome			
Liver fire syndrome	Vertigo, headache, facial flushing with perspiration, conjunctival congestion, bitter taste in the mouth, thirst, irritability and restlessness, wiry-rapid-powerful pulse or powerful cunkou pulse alone, or wiry and long pulse even well beyond the cunkou pulse	Calming liver and suppressing liveryang hyperactivity	Tianma Gouteng decoction, Zhengan Xifeng decoction, Jianling decoction, and Longdan Xiegan decoction
Heart fire syndrome	Facial flushing with perspiration, bitter taste in the mouth, thirst, insomnia, red tip of the tongue, and rapid pulse	Clearing heart fire	Zhi-zi-chi decoction, Sanhuang Xiexin decoction, and Huanglian Jiedu decoction
Stomach fire syndrome and intestine fire syndrome	Dry mouth, thirst with desire for cold drinks, easy to starve, foul breath, abdominal distension and pain, smelly stool, constipation, red tongue, yellow dry fur, right guan pulse powerful alone, or strength and deep-hidden-powerful pulse	Clearing stomach-intestine fire, promoting digestion, relaxing bowels, and relieving constipation	Da Chai Hu decoction, Baohe pill, Baihu decoction, Houpu Dahuang decoction, Gegen Qinlian decoction, and Zeng Ye decoction

Phlegm-fluid retention syndrome			
Phlegm and dampness syndrome	Obesity, dizziness, sticky mouth, thirst without a desire to drink, chest distress, nausea, vomiting, anorexia, abdominal distension, loose stools, sleepiness, greasy tongue coating, and slippery pulse	Dispelling phlegm and eliminating dampness	Erchen decoction, Pingwei powder, Wendan decoction, Banxia Baizhu Tianma decoction, and Xiao Xianxiong decoction
Fluid retention syndrome	Dizziness aggravated by change in body position, thirst without a desire to drink or not being thirsty, chest distress, palpitation, gastric distension, abdominal distension, poor appetite, lumbar heaviness, weakness and heaviness in the lower extremities, edema, daytime sleepiness, abnormal leucorrhea, dysuria, greasy fur, swollen tongue, and deep pulse	Dissipating excessive fluid	Banxia baizhu tianma decoction, Wuling powder, Zhuling decoction, Zexie decoction, and Fuling Guizhi Baizhu Gancao decoction

Deficiency syndrome			
Spleen deficiency syndrome	Fatigue, shortness of breath, stomach pain, poor appetite, abdominal distension, and loose stools	Reinforcing spleen	Fuling Guizhi Baizhu Gancao decoction, Si jun Zi decoction, and Liu Jun Zi decoction
Kidney deficiency syndrome	Tiredness in the loins and legs, tinnitus and dizziness, sexual dysfunction, dysuria, weakness and fatigue, and weak chi pulse	Reinforcing kidney	Liuwei Dihuang pill and Shenqi pill

Each CHM under the classical formula is composed of multiple herbs.

**Table 2:** The characteristics of systematic reviews and meta-analysis of CM for hypertension.

Intervention	Title	Authors	Year published	Trials	Participants included	Authors' comments
	Meta-analysis of effectiveness of antihypertension of 442 traditional Chinese herbal decoctions	Ding and Zhou [43]	2012	7	808	Limited evidence suggested that total effective rate and efficiency of CM are lower than those of single WM as for BP controlling. But owing to lack of data from high-quality RCT, the efficacy need to be further studied.
	Quantitative analysis of clinical controlled trials of traditional Chinese medicine and systematic evaluation of randomized controlled trials involving traditional Chinese medicine for essential hypertension	Hu [44]	2009	24	1660	CM combined with WM showed better results than WM for treating hypertension. However, due to the generally low quality of the trials, large sample, multicenter double blind RCTs with strict design are warranted.
	Meta-analysis of traditional Chinese medicine for essential hypertension	Ren et al. [45]	2006	11	1010	CM may be beneficial to reduce BP in patients with hypertension.
Chinese Herbal medicine	Systematic review and meta-analysis of Tianma Gouteng Yin combined with enalapril for essential hypertension	Dong et al. [46]	2011	6	543	Tianma Gouteng Yin combined with enalapril showed additional better effects than enalapril for hypertension. No serious adverse event is reported. Due to the low methodological quality and potential bias of trials, large-sample, multicenter, randomized, double-blind, controlled trials are warranted.
	Tianma Gouteng Yin Formula for treating primary hypertension^E^	Zhang et al. [47]	2012	0	0	The review could not find any randomized controlled clinical trials that compared Tianma Gouteng Yin Formula (TGYF) to placebo or no treatment. The authors cannot draw a conclusion that TGYF may be beneficial for hypertension. Well-designed randomized controlled studies need to be conducted and published.
	Systematic review of clinical evidence about calm the liver and subdue yang therapy on the hypertension disease with the syndrome of upper hyperactivity of liver yang	Xu and Li [48]	2012	8	944	The calm the liver and subdue yang therapy for treating hypertension disease with syndrome of upper hyperactivity of liver yang has curative effect and high safety. However, owing to lack of data from high-quality RCT and potential publication bias, the positive findings should be interpreted conservatively.
	Systematic review of replenishing kidney *qi* method for essential hypertension with kidney *qi* deficiency syndrome	Shi and Zhang [49]	2012	5	457	The replenishing kidney *qi* therapy for treating hypertension with kidney *qi* deficiency syndrome has curative effect and high safety. High-quality and large-scale RCTs are needed to further prove the results of the study because of the low quality of the included studies.
	Systematic review on treatment of essential hypertension from spleen and kidney deficiency	Liu and Li [50]	2011	15	1661	Treatment of essential hypertension from the spleen and kidney deficiency was effective, and the level of safety is reliable. However, the quality of most trials was low.
	Effects of Chinese medicine on elderly isolated systolic hypertension: a meta-analysis	Li and Yang [51]	2012	17	1323	Chinese medicine is effective on treating isolated systolic hypertension of the old, as well as reducing symptoms and pulse pressure.

Acupuncture	The effect of acupuncture therapy on essential hypertension: a systematic review of long-term effect	Zhao et al. [52]	2011	18	1460	Although it shows a tendency that acupuncture can improve the conditions of essential hypertension, a reliable conclusion cannot be drawn from the present data because of the defects in methodological quality and insufficient numbers of trials. It is necessary to perform more multicentral RCTs of high quality in the future.

Moxibustion	Moxibustion for hypertension: a systematic review^E^	Kim et al. [53]	2010	4	240	There is insufficient evidence to suggest that moxibustion is an effective treatment for hypertension. Rigorously designed trials are warranted to answer the many remaining questions.

Cupping	Cupping for hypertension: a systematic review^E^	Lee et al. [54]	2010	2	76	The evidence is not significantly convincing to suggest that cupping is effective for treating hypertension. Further research is required to investigate whether it generates any specific effects for that condition.

Qigong	Qigong for hypertension: a systematic review of randomized clinical trials^E^	Lee et al. [55]	2007	12	1332	There is some encouraging evidence of qigong for lowering SBP, but the conclusiveness of these findings is limited. Rigorously designed trials are warranted to confirm these results.
	Clinical effect of Qigong practice on essential hypertension: a meta-analysis of randomized controlled trials^E^	Guo et al. [56]	2008	9	908	Self-practiced qigong for less than 1 year is better in decreasing BP in patients with essential hypertension than in no-treatment controls, but is not superior to that in active controls. More methodologically strict studies are needed to prove real clinical benefits of qigong and to explore its potential mechanism.

Tai Chi	Systematic review of Tai Chi for essential hypertension	Li and Xu [57]	2011	5	318	Tai Chi is effective on treating essential hypertension. However, different exercise time of Tai Chi has an impact on hypertensive patients. More RCTs of high quality are warranted to prove benefits of Tai Chi on hypertensive patients with different stages.

CM: Chinese medicine; WM: Western medicine; RCT: randomized controlled trial; BP: blood pressure; SBP: systolic blood pressure; and E: in English.
